# Preventing, screening, and intervening in youth substance use: examining implementation of SBIRT in diverse settings

**DOI:** 10.1186/1940-0640-10-S2-O28

**Published:** 2015-09-24

**Authors:** Cori Sheedy, Dana Hunt

**Affiliations:** 1 US Health, Abt Associates, Cambridge, USA

## Background

The Screening, Brief Intervention, and Referral to Treatment (SBIRT) model has brought positive outcomes in addressing adult substance use, but its effectiveness during adolescence-a time of high-risk behavior-while promising, is less known. Under a three-year grant from the Conrad N. Hilton Foundation, Abt is evaluating more than two dozen health, school, and community-based settings providing SBIRT services to youth and young adults funded through the Foundation's Youth Substance Use Prevention and Early Intervention Strategic Initiative.

## Material and methods

Grantees' quarterly reporting on the Initiative's three objectives (expand training, increase implementation, and promote the evidence base) and individualized RE-AIM measures (Reach, Effectiveness, Adoption, Implementation, and Maintenance) and key informant interviews with grant programs' project directors using a structured interview guide provide early results on training and implementation challenges and successes, as well as initial results on screening tools and brief intervention methodologies.

## Results

In any setting, implementing an innovation is difficult, time-consuming, and requires following specific steps that account for the intricacies of the environment, while maintaining fidelity to the evidence base. This is even more difficult with varying models of implementation, settings to which it is being implemented, and the black box of “brief intervention.” Grantees report key factors that cross implementation setting: engaging the right people, including an innovation early adopter or champion; modifying electronic health record and/or incorporating SBIRT into the medical, school, or community “record”; and ensuring confidentiality/privacy of youth.

## Conclusions

Physicians' judgments, rather than validated screening tools, tend to drive interventions for youth and young people. The study's findings will be used to improve delivery systems aimed at youth, strengthen the evidence base for prevention and treatment, and improve school and workplace success as it helps policymakers, funders, and practitioners better understand substance use and its impact. Additional research will be conducted that reports on training model's effectiveness and outcomes related to varying models of SBIRT implementation.

